# Factors Influencing Adherence to Tuberculosis Treatment in the Ketu North District of the Volta Region, Ghana

**DOI:** 10.1155/2021/6685039

**Published:** 2021-03-30

**Authors:** Eyram Dogah, Mark Aviisah, Da-Ama Mawulom Kuatewo, Godsway Edem Kpene, Sylvester Yao Lokpo, Florence Shine Edziah

**Affiliations:** ^1^Disease Control Unit, Ketu North Municipal Health Directorate, Ghana Health Service, Dzodze, Volta Region, Ghana; ^2^Department of Health Policy, Planning and Management, School of Public Health, University of Health and Allied Sciences, Hohoe, Ghana; ^3^Hohoe Municipal Health Directorate, Ghana Health Service, Hohoe, Volta Region, Ghana; ^4^Department of Medical Laboratory Sciences, School of Allied Health Sciences, University of Health and Allied Sciences, Ho, Ghana

## Abstract

Annually, ten million cases of tuberculosis (TB) and about 1.8 million mortalities are recorded. Adherence to TB treatment not only reduces death outcomes but prevents prolonged sickness, transmission to others, and the development of multidrug-resistant TB. This study is aimed at determining the rate of treatment adherence, knowledge of TB infection, and the possible factors influencing adherence to TB treatment in the Ketu North District in the Volta Region of Ghana. A cross-sectional study design was employed. A semistructured questionnaire was used to obtain data from respondents. Adherence to TB treatment and knowledge level about TB infection were assessed. A Chi-square test analysis was used to determine the variables that were associated with treatment adherence. Logistic regression analysis was used to determine potential factors that contribute to treatment adherence. A total of 125 TB registrants were enrolled in the study. The majority (102 (81.6%)) adhered to the TB treatment regimen. However, the level of knowledge about night sweat being a symptom of TB infection was relatively low (78 (62.4%)). Logistic regression analysis revealed that the male gender was about three times more likely (OR = 2.978, 95%CI = 1.173‐7.561; *p* = 0.022) to be associated with adherence to TB treatment. However, food availability (OR = 2.208, 95% CI (0.848-5.753); *p* = 0.10) and household size (OR = 0.538, 95% CI (0.195-1.483); *p* = 0.23) were not significantly associated with treatment adherence. In this study, adherence to TB treatment and the knowledge level of TB infection were high. However, the knowledge level of night sweat being a symptom of TB infection was relatively low. Being a male was significantly associated with treatment adherence. An intensified health education on the symptoms of TB infection is therefore recommended.

## 1. Background

Tuberculosis (TB) is an infectious yet preventable and curable disease caused by *Mycobacterium tuberculosis* [1]. TB affects the lung (pulmonary TB), but it can also affect other parts of the body (extrapulmonary TB). Global reports indicate that about 10 million people were infected with TB in 2019, with some African countries among the leading nations that contributed to the newest TB cases [2]. TB treatment is aimed at ensuring cure for the individual, minimizing the risk of death and disability, and reducing transmission of *M. tuberculosis* [3]. In view of this, the WHO introduced the Directly Observed Treatment (DOT) short-course strategy in 1998 [4] for the purposes of case detection, management, and monitoring [5]. The DOT strategy was also to help improve adherence and enhance treatment outcomes and thus prevent the development of drug resistance [6]. A more recent global target adopted in the United Nations Sustainable Development Goals (SDGs) is to end the TB epidemic by 2030 [7]. Although there appears to be a decline in the annual incident rate, current records show that an estimated 206,030 people worldwide have developed multidrug- or rifampicin-resistant TB (MDR/RR-TB), underscoring the fact that TB infection and control remain a major global public health concern [7].

The WHO defines adherence as the degree to which the person's behavior corresponds with the agreed recommendations from a health care provider [8]. For patients to be successful in TB treatment, they must take the anti-TB drugs for at least six months uninterrupted [3]. However, about half of all TB patients worldwide fail to complete the treatment regimen suggesting the other half to be nonadherent to TB treatment [9]. Some factors proposed to contribute to nonadherence to TB treatment include a false sense of recovery before completing treatment, side effects of the drugs, and ignorance, as well as depression, forgetfulness, and failure to procure medicines from the health facility [5]. Preference for traditional medicine, economic, and geographical difficulties are other suggested factors influencing nonadherence [10].

In West Africa, like most countries in the subregion, Ghana continues to record high TB infection rates as 13,978 new cases were reported in 2018, constituting an incident rate of 32% [11, 12]. The attempt to halt the high prevalence led the country's health ministry to implement a five-year strategic plan (2015-2020) aimed at improving treatment success rate through improved quality clinical care and community TB care [13]. Despite this initiative, records available in the Volta Region show that TB cases remain high, with about 60 and 58.2 TB cases per 100,000 population estimated in 2016 and 2017, respectively [11]. Moreover, there is a dearth of literature on the adherence rate, knowledge level of TB infection, and factors influencing adherence to TB treatment in Ghana, although a previous study in the Eastern Region of Ghana reported a prevalence of 22% noncompliance to treatment among TB patients [14]. Hence, in this study, we aimed to provide information on the rate of treatment adherence and identify factors influencing treatment adherence in the Ketu North District of the Volta Region.

## 2. Materials and Methods

### 2.1. Study Design

A cross-sectional study design was employed in this study.

### 2.2. Study Site Description

Dzodze is the capital of Ketu North District in the Volta Region. Ketu North District shares a boundary with Keta Municipality to the southwest, Akatsi North District to the north, and the Republic of Togo to the east and is bounded by Akatsi South to the east. Ewe is the widely spoken language. Ketu North District has a population of 99,913 with a total number of 26,437 households. The district has an average household of 3.7 persons per household according to the Ghana Statistical Service [15]. There are two hospitals, two clinics, seven health centers, four subdistricts, and thirty-seven CHPS zones. Skilled agriculture, forestry, and fishing constitute the most extensive (47.1%) occupational group, followed by those in craft and related trades (19.8%), and services and sales workers (16.7%). The methods of diagnosing TB in the district include the Acid-Fast Bacilli (AFB) smear microscopy and culture. When a patient is diagnosed with TB, the treatment takes a minimum of six months. The district employs a DOT strategy where a family member or caretaker or a health professional supervises patients to take their medications.

### 2.3. Study Population

The study population included patients whose records were found in the TB register between November 2014 and August 2016. A health care provider at the facility helped to identify those in the TB register that met the criteria for participation in the study. These patients were informed on the rationale of the study, referred for participation, and contact information was given to ease tracing.

### 2.4. Inclusion and Exclusion Criteria

The study subjects included registered patients with pulmonary and or extrapulmonary TB, traceable, and consented to be part of the study. However, patients that were drug abusers, were terminally ill, did not give informed consent, and were not traceable were excluded from the study.

### 2.5. Sample Size Determination

A minimum sample size of 123 was calculated employing the Raosoft online sample size calculator (http://www.raosoft.com/samplesize.html). This was obtained from a total of 180 TB-registered clients, at a 95% confidence interval, 50% response rate, and 5% margin of error.

### 2.6. Sampling Technique

A convenient and purposive sampling technique was employed. A qualified health worker at the facility helped to identify the 125 respondents who met the inclusion criteria and were reachable.

### 2.7. Data Collection Procedures

Patients were well informed about the rationale of the study and the consent provided. A structured questionnaire was used to capture data on demographic characteristics and socioeconomic parameters and to assess participants' knowledge about TB, TB treatment, and factors that influence adherence. For confidentiality and anonymity, the names of the respondents were not included in the questionnaire.

### 2.8. Data Analysis

Data were entered into Microsoft Excel version 13, cleaned, and exported to Social Package for Social Sciences (SPSS) version 23.00 (SPSS Inc., Chicago, USA (http://www.spss.com)) for analysis. Chi-square test and logistic regression analyses were performed to determine the association between variables and adherence and potential factors that could influence adherence to TB treatment, respectively. Variable with *p* values less than or close to 0.05 after a Chi-square test analysis were included in the logistic regression analysis. At a 95% level of confidence, a *p* value of less than 0.05 was considered statistically significant.

### 2.9. Assessment of Knowledge Levels and Definition of Adherence to TB Treatment

The knowledge level of respondents was assessed on TB symptoms, mode of transmission, and treatment duration. The symptoms include coughing, chest pain, weight loss, and night sweats. A right response was regarded as good knowledge (score = 1) while a wrong response was regarded as poor knowledge (score = 0). The total score for an individual below the index score of 4 was regarded as inadequate knowledge while scores above the index score of 4 were regarded as adequate knowledge. Adherence was defined as self-report of having completed TB treatment plus documented evidence from the TB register of having completed treatment regimen as prescribed by the clinician.

### 2.10. Ethical Consideration

The study was approved by the Ethical Review Committee of Ghana Health Service, Research, and Development Division. Written approval was obtained from Ketu North Health Directorate and St. Anthony Catholic Hospital. Informed consent was obtained from respondents before the interview.

## 3. Results

The majority of the study participants were males (76 (60.80%)) and Christians (99 (79.2%)), and 42 (33.6%) were between 30 and 39 years. At the time of this study, the majority of the study participants were married (74 (59.2%)) and 116 (92.8%) were employed while 54 (42.2%) attained primary education whereas most respondents were of the Ewe ethnicity 113 (90.40%) (Table 1).

From Table 2, it was observed that all respondents exhibited good knowledge regarding coughing as a symptom. The majority of study respondents demonstrated good knowledge on the mode of TB transmission 100 (80%), duration of treatment (124 (99.2%)), chest pain (117 (93.6)), and weight loss (102 (81.6)) as well as night sweats (78 (62.4)) and coughing 125 (100%) as symptoms of TB infection. When participants were asked when it was appropriate to stop treatment, 121 (96.8) indicated that one needed to continue treatment even when they feel well while 124 (99.2) said it should only be halted when a health worker asks you to stop.

The Chi-square test analysis showed that gender was a significant factor associated with treatment adherence. However, educational level, having treatment supporter, staff attitude, self-perceived wellness, food availability, being on other medication, and household size were not significantly associated with treatment adherence (Table 3).

Males were about thrice as likely to adhere to TB treatment compared to females (OR = 2.978, 95%CI = 1.173‐7.561; *p* = 0.022). However, food availability (OR = 2.208, 95% CI (0.848-5.753); *p* = 0.10) and household size (OR = 0.538, 95% CI (0.195-1.483); *p* = 0.23) did not significantly influence adherence to TB treatment (Table 4).

## 4. Discussion

Adherence to TB treatment decreases the risk of drug resistance, prolonged infection, or even death [16]. In the current study, we provide empirical data to the literature on adherence of TB patients to treatment in the Ketu North District in the Volta Region of Ghana, which hitherto was lacking. The adherence rate (81.6%) in this study (Table 3) was higher than that recorded in Suhum in the Eastern Region of Ghana (63%) [14] but lower than rates reported in Tanzania (95.7%) [17] and Ethiopia (90.0%) [18]. Compared to the earlier reports in Suhum, the plausible explanations to the higher adherence rate in our study include increased awareness of TB infection via effective health education in the district and the relatively greater proportion of volunteered treatment supporters (84.80%). Moreover, differences in methodology including study design, type of sampling technique, sample size, and definition of adherence could account for the dissimilarities in the adherence rates between our study and those recorded in Tanzania and Ethiopia.

It is suggested that patients' knowledge about a disease influences treatment adherence and outcome [19]. A previous study in the Sekondi-Takoradi Metropolis observed that failure to recognize symptoms due to TB and stigma negatively affect patients' adherence to treatment [20]. In our study, the overall knowledge level of TB infection was high (119 (95.2%)) (Table 3). Moreover, a greater proportion of the study respondents demonstrated good knowledge about the mode of transmission (100 (80.0%)), duration of treatment (124 (99.2%)), and symptoms of TB including coughing (125 (100.0%)), chest pain (117 (93.6%)), and weight loss (102 (81.6%)) (Table 2). Indeed, having adequate knowledge about a disease could translate into improved adherence because the patient is less likely to halt treatment during the treatment period [19]. This appears to be the case in this study where the high knowledge level exhibited by the study respondents could account for the high adherence rate recorded. However, the level of knowledge about night sweat being a symptom of TB infection in this study seems to be relatively low (78 (62.4%)) ((Table 2), indicating that more should be done to intensify education on the symptoms of TB infection in the district. This finding is consistent with a prior study in Morocco where only 17.2% of respondents had displayed good knowledge about the causative agent of TB [21] and Equatorial Guinea where 16.84% knew about the mode of transmission [22].

An earlier report in Ghana suggested that patients' compliance with medication was not influenced by their background characteristics [14]. In our study, however, we observed gender to be significantly associated with treatment adherence where the male respondents were about three times more likely to adhere to TB treatment (OR = 2.978, 95%CI = 1.173‐7.561; *p* = 0.022) compared to their female counterparts (Table 4). No specific reason can be adduced from the current study. However, further studies are required to confirm this finding and to unravel the possible factors that could contribute to male preponderance to TB treatment adherence in the district. In contrast, females were twice more likely to adhere to TB treatment compared to their male peers in the Tanzanian study [17].

Salifu and Eliason [23] in their study to understand patients' perspectives of barriers and enablers to TB treatment observed that food availability and medication-related side effects impacted negatively on treatment adherence. In contrast, we did not find a significant association between food availability and treatment adherence (OR =2.208, 95% CI (0.848-5.753); *p* = 0.10) (Table 4), although the adherence rates were higher among those who had food available compared to those who did not (Table 3). The feeling of uneasiness after taking medication on an empty stomach or with inadequate food has been reported previously in individuals who had defaulted in TB treatment [23]. Indeed, having a large family size is believed to impact negatively on household food security [24]. Therefore, this impact could also be felt by individuals undergoing TB treatment in such large households. In our study, however, no significant association was observed between household size and treatment adherence (OR = 0.538, 95% CI (0.195-1.483); *p* = 0.23) (Table 4).

The present study is limited by the relatively small sample size; hence, a larger sample size should be used in future studies in the district. Due to the cross-sectional study design employed in this study, cause and effect relationships cannot be established. Notwithstanding, the findings of this study address an important gap in the literature regarding the adherence rate, knowledge level, and possible factors that could contribute to adherence to TB treatment in the Ketu North District in the Volta Region of Ghana.

## 5. Conclusion

In this study, adherence to TB treatment and the knowledge level of TB infection were high. However, the level of knowledge about night sweat being a symptom of TB infection was relatively low. Being a male was significantly associated with treatment adherence while food availability and household size were not significantly associated with treatment adherence. We recommend an intensified health education on the symptoms of TB infection in the district.

## Figures and Tables

**Table 1:** Demographic characteristics of the study respondents.

Parameter	Frequency	Percentage
Age (years)	40.23 ± 8.79^∗^	
Age category (years)		
20–29	18	14.4
30–39	42	33.6
40–49	41	32.8
50–59	24	19.2
Gender		
Female	49	39.2
Male	76	60.8
Educational status		
None	20	16.0
Primary	54	42.2
Secondary	43	34.4
Tertiary	8	6.4
Religion		
Christianity	99	79.2
Islam	5	4
Traditional	16	12.8
Others	5	4
Marital status		
Single	16	12.8
Cohabiting	15	12
Married	74	59.2
Separated	11	8.8
Widowed	9	7.2
Ethnicity		
Akan	2	1.6
Ewe	113	90.4
Others	10	8
Employment status		
Employed	116	92.8
Unemployed	9	7.2

Data are presented as figures and percentages. ∗ is expressed as mean ± standard deviation.

**Table 2:** Respondent's knowledge of TB symptoms, transmission, and treatment duration.

Parameter	Knowledge level	
	Poor	Good
Mode of transmission of TB	25 (20.0)	100 (80.0)
Duration of treatment	1 (0.8)	124 (99.2)
*Symptoms of tuberculosis*		
Coughing	0 (0.0)	125 (100.0)
Chest pain	8 (6.4)	117 (93.6)
Weight loss	23 (18.4)	102 (81.6)
Night sweat	47 (37.6)	78 (62.4)
*When to halt treatment*		
When the individual feels well	4 (3.2)	121 (96.8)
When a health worker asks you to stop	1 (0.8)	124 (99.2)
*Overall knowledge level on TB infection*	*Inadequate*	*Adequate*
	6 (4.8)	119 (95.2)

Data is presented frequencies and percentages in parentheses.

**Table 3:** Chi-square test analysis of factors associated with treatment adherence.

Parameter	Treatment adherence			*p* value
	Total (125 (100))^a^	No (23 (18.4))^b^	Yes (102 (81.6))^b^	
Gender				
Female	49 (39.20)	14 (28.57)	35 (71.43)	0.02
Male	76 (60.80)	9 (11.84)	67 (88.16)	
Age category (years)				
<40	60 (48.00)	13 (21.66)	47 (78.34)	0.49
≥40	65 (52.00)	10 (15.38)	55 (84.52)	
Educational level				
Up to primary	74 (59.20)	14 (18.92)	60 (81.08)	0.86
Above primary	51 (40.80)	9 (17.65)	42 (82.35)	
Treatment supporter				
No	19 (15.20)	4 (21.05)	15 (78.95)	0.75
Yes	106 (84.80)	19 (17.92)	87 (82.08)	
Staff attitude				
Friendly	99 (79.20)	18 (18.18)	81 (81.82)	0.90
Nonfriendly	26 (20.80)	5 (19.23)	21 (80.77)	
Self-perceived wellness after initiating treatment				
<2 months	23 (18.40)	6 (26.09)	17 (73.91)	0.29
2-4 months	102 (81.60)	17 (16.67)	85 (83.33)	
On other medications				
No	94 (75.20)	15 (15.96)	79 (84.04)	0.22
Yes	31 (24.80)	8 (25.81)	23 (74.19)	
Food availability				
Available	93 (74.40)	14 (15.05)	79 (84.95)	0.10
Not available	32 (25.60)	9 (28.13)	23 (71.88)	
Household size				
1–2	10 (8.00)	4 (40.00)	6 (60.00)	0.09
3–4	35 (28.00)	8 (22.86)	27 (77.14)	
>4	80 (64.00)	11 (13.75)	69 (86.25)	

Data is presented as figure and percentages in parentheses. *p* value significant at <0.05. ^a^Percentages are calculated columnwise. ^b^Percentages are calculated rowwise.

**Table 4:** Logistic regression analysis of factors influencing treatment adherence.

Parameter	OR	95% CI	*p* value
Gender			
Female	Ref		
Male	2.978	1.173–7.561	0.02
Food availability			
Available	Ref		
Not available	2.208	0.848–5.753	0.10
Household size			
1–2	Ref		
3–4	0.239	0.058–0.986	0.05
>4	0.538	0.195–1.483	0.23

Data are presented as odds ratio (OR) and 95% confidence interval (CI). *p* value significant at <0.05.

## Data Availability

The datasets used during the current study are available from the corresponding author on request.

## References

[1] WHO (2015). *Global Tuberculosis Report*.

[2] WHO (2019). *Tuberculosis Secondary Tuberculosis*.

[3] CDC (2011). *Tuberculosis (TB) Fact Sheet Secondary Tuberculosis (TB) Fact Sheet*.

[4] Ubajaka C. F., Azuike E. C., Ugoji J. O. (2015). Adherence to drug medications amongst tuberculosis patients in a tertiary health institution in south east Nigeria. *International Journal of Clinical Medicine*.

[5] Kaona F. A. D., Tuba M., Siziya S., Sikaona L. (2004). An assessment of factors contributing to treatment adherence and knowledge of TB transmission among patients on TB treatment. *BMC Public Health*.

[6] Yao S., Huang W.-H., van den Hof S. (2011). Treatment adherence among sputum smear-positive pulmonary tuberculosis patients in mountainous areas in China. *BMC Health Services Research*.

[7] WHO (2019). *Global Tuberculosis Report 2018*.

[8] Dobbels F., Van Damme-Lombaert R., Vanhaecke J., De Geest S. (2005). Growing pains: non-adherence with the immunosuppressive regimen in adolescent transplant recipients. *Pediatric transplantation*.

[9] Munro S. A., Lewin S. A., Smith H. J., Engel M. E., Fretheim A., Volmink J. (2007). Patient adherence to tuberculosis treatment: a systematic review of qualitative research. *PLoS Medicine*.

[10] Rondags A., Himawan A. B., Metsemakers J. F., Kristina T. N. (2014). Factors influencing non-adherence to tuberculosis treatment in Jepara, Central Java, Indonesia. *The Southeast Asian Journal of Tropical Medicine and Public Health*.

[11] Report G. (2018). *The Health Sector in Ghana Facts and Figures*.

[12] Team DHM (2015). *Annual Year Rewiew of Ketu North District Health Directorate*.

[13] Ministry of Health G (2015). *The National Tuberculosis Health Sector Strategic Plan for Ghana*.

[14] Danso E., Addo I. Y., Ampomah I. G. (2015). Patients’ compliance with tuberculosis medication in Ghana: evidence from a periurban community. *Advances in Public Health*.

[15] GSS (2010). *Ghana Statistical Services*.

[16] Tesfahuneygn G., Medhin G., Legesse M. (2015). Adherence to anti-tuberculosis treatment and treatment outcomes among tuberculosis patients in Alamata District, northeast Ethiopia. *BMC Research Notes*.

[17] Mkopi A., Range N., Lwilla F. (2012). Adherence to tuberculosis therapy among patients receiving home-based directly observed treatment: evidence from the United Republic of Tanzania. *PloS one*.

[18] Tola H. H., Holakouie-Naieni K., Tesfaye E., Mansournia M. A., Yaseri M. (2019). Prevalence of tuberculosis treatment non-adherence in Ethiopia: a systematic review and meta-analysis. *The International Journal of Tuberculosis and Lung Disease*.

[19] Chani K. (2010). *Factors Affecting Compliance to Tuberculosis Treatment in Andara Kavango Region Namibia*.

[20] Dodor E. A. (2012). The feelings and experiences of patients with tuberculosis in the Sekondi-Takoradi Metropolitan district: implications for TB control efforts. *Ghana Medical Journal*.

[21] Tachfouti N., Slama K., Berraho M., Nejjari C. (2012). The impact of knowledge and attitudes on adherence to tuberculosis treatment: a case-control study in a Moroccan region. *The Pan African Medical Journal*.

[22] Fagundez G., Perez-Freixo H., Eyene J. (2016). Treatment adherence of tuberculosis patients attending two reference units in Equatorial Guinea. *PloS one*.

[23] Salifu Y., Eliason C. (2017). Tuberculosis treatment adherence in Ghana: patients' perspectives of barriers and enablers to treatment. *NUMID HORIZON: An International Journal of Nursing and Midwifery*.

[24] Olayemi A. O. (2012). Effects of family size on household food security in Osun state, Nigeria. *Asian Journal of Agriculture and Rural Development*.

